# Pre-exposure Prophylaxis as HIV Prevention in High Risk Adolescents

**Published:** 2018-01-16

**Authors:** Megan E. Gray, Sheela V. Shenoi, Rebecca Dillingham

**Affiliations:** 1 Division of Infectious Diseases and International Health. University of Virginia, Charlottesville, VA, USA; 2 Assistant Professor of Medicine. Yale University, New Haven, CT, USA; 3 Director of the Center for Global Health, Associate Professor of Medicine. Division of Infectious Diseases and International Health. University of Virginia, Charlottesville, VA, USA

**Keywords:** HIV, AIDS, Adolescents, Prevention, Pre-exposure prophylaxis

## Introduction

A recently published review of the pediatric Human Immunodeficiency Virus (HIV) continuum of care in Pediatric Clinics of North America discussed methods of HIV prevention for children and adolescents^1^. HIV pre-exposure prophylaxis (PrEP), the use of antiretroviral medications in uninfected persons to prevent HIV infection, was mentioned in the review. The use of PrEP in high risk adolescent populations is an emerging practice. Significant knowledge gaps remain, but the available data to guide current practice is growing due to the vigorous state of PrEP research. A dedicated discussion of PrEP for select adolescents is merited.

Adolescence refers to the period of transition between childhood and adulthood, which begins with puberty and is characterized by psychological, cognitive, social, socioeconomic and sexual development. The exact ages that encompass adolescence are arguably indistinct, though the age range of 10–19 is used by the World Health Organization (WHO)^2,3^. Most of the research done to establish efficacy and safety of PrEP has been conducted with predominantly adult populations, or those over the age of 18. Few PrEP studies exclusively among adolescents have been done, though more studies are underway^4–7^.

The first study evaluating oral PrEP among men who have sex with men (MSM) was published in 2010 and showed efficacy of once daily tenofovir disoproxil fumarate (TDF) and emtricitabine (FTC)^8^. Several studies followed^9–11^, all including patients >18yo, leading the United States Food and Drug Administration (FDA) to approve TDF-FTC (trade name Truvada^®^) for PrEP use in 2012. Labeling specifies that use is for adults at high risk for HIV, though clarification on what age is considered adulthood is not given^12^. While TDF-FTC is presently the only approved formulation of PrEP, several formulations have been studied, including intravaginal gels^13,14^, monthly intravaginal rings^15^, long acting intramuscular injections^16^, and long-acting subcutaneously implanted polymer devices^17^.

## Consensus Guidelines

Currently, there are no countries that have approved the use of oral PrEP for adolescents^4^. However, several organizations have issued guidelines regarding PrEP in sexually active adolescent populations. The WHO revised their guidelines in 2015 to recommend PrEP to any person with substantial risk of HIV infection as a part of a comprehensive prevention bundle. This was a change from the WHO’s 2012 guidelines, which recommended PrEP only for MSM, transgender people, and serodiscordant couples. The WHO defines substantial risk as HIV incidence of greater than 3 per 100 person-years in the absence of PrEP usage^2^. This risk may be difficult to assess on an individual or sub-population basis. However, there are certain groups at elevated risk and country-dependent vulnerable populations that have substantial risk based on available epidemiologic data.

The United States Centers for Disease Control and Prevention (CDC) briefly discusses the use of PrEP in adolescent minor populations within their 2014 PrEP clinical practice guideline, recommending that the risks and benefits should be carefully considered in the framework of applicable laws and regulations as there is insufficient data on the use of PrEP in adolescent populations^18^. The U.S. President’s Emergency Plan for AIDS Relief (PEPFAR) developed their own PrEP guidelines in 2015, which recommended prioritizing PrEP for adolescent girls and young women (AGYW), ages 15–24, in the ten countries targeted by the PEPFAR DREAMS initiative^19^.

United Nations International Children’s Emergency Fund (UNICEF) and New York State Department of Health AIDS Institute both have published reports summarizing meetings that discuss challenges in and priorities for implementation of PrEP in adolescent populations^20,21^. Challenges and concerns raised by a panel of young advocates and youth taking PrEP at the UNICEF meeting are presented in Table 1^21^.

## PrEP in High Risk Groups and Vulnerable Populations

### Adolescent Girls and Young Women

Globally, AGYW are at the highest risk for HIV acquisition. In sub-Saharan Africa, AGYW are up to four times more likely to be infected with HIV than their male equivalents due to sociocultural issues including gender based violence, child marriage, and early pregnancy with subsequent low secondary school completion rates and lower socioeconomic independence^19^. The PEPFAR DREAMS initiative aims to reduce HIV among AGYW in sub-Saharan Africa. Over 50% of new HIV infections globally occur in this region, with AGYW accounting for a third of these^19^.

Several randomized clinical trials have been performed among women in sub-Saharan Africa, including young women. The FEM-PrEP^11^ and VOICE^22^ trials recruited women over the age of 17 and compared oral PrEP to placebo or to placebo and TDF vaginal gel, respectively. Unfortunately, both trials showed no effect of PrEP due to poor adherence. Younger, unmarried women had poorer adherence^22^. A South African study (ADAPT) evaluated daily oral TDF-FTC vs two different methods of on-demand PrEP, taken only at times of sexual activity, among women 18 years or older. Daily PrEP led to increased coverage of sex events and better adherence, with no significant difference in adherence between age groups^23^. This is the first study showing satisfactory PrEP adherence among young women in sub-Saharan Africa. No studies have evaluated the use of PrEP in adolescent girls and while their HIV risk is similar to that of young women, adolescent girls may face additional age-related barriers to PrEP, such as intensified stigma and disapproval from parents or peers.

### Male adolescents and young men who have sex with men

In the United States, 27% of new HIV diagnoses among MSM occur between the ages of 13 and 24. Of all new HIV diagnoses in this age group, 92% are gay or bisexual^24^, which led to the first and only published study to date assessing feasibility of daily oral PrEP in an exclusively adolescent population. Trial ATN-113 was published in September 2017. It included adolescent MSM from the ages of 15 and 17 and showed daily TDF-FTC to be well-tolerated. Drug levels were detected in more than 95% of participants in the first 12 weeks, but detectable levels decreased thereafter, correlating with a scheduled decrease in frequency of follow-up visits from monthly to quarterly^7^. A similar trend in adherence was observed in trial ATN-110, among MSM ages 18–22^25^. On-demand PrEP has demonstrated 97% efficacy in reducing HIV incidence among transgender women and MSM ages 18 or older^26^, and may be a future option for high risk male adolescents, but data are lacking.

### Other risk groups

Adolescents who inject drugs are at substantial risk of HIV infection due to shared injection equipment, disinhibited sexual behavior, and transactional sex. There has only been one randomized control trial evaluating PrEP among people who inject drugs, which demonstrated good efficacy, but did not include adolescents^27^.

HIV uninfected long-term sexual partners of HIV infected persons are a high risk group that can benefit from PrEP. The Partners PrEP study found the efficacy of oral PrEP in preventing HIV transmission to male and female partners to be lower in the 18–24 age group compared to those 25 years or older, possibly due to differences in adherence. However, those in the younger age group still experienced a 41–72% risk reduction^9^. PrEP for serodiscordant couples during adolescence is likely less common due to more transient sexual relationships during this period, one notable exception being age-discordant relationships among AGYW in sub-Saharan Africa^19^.

Several additional studies evaluating PrEP use in adolescent populations are underway^4,28,29^, including a study evaluating PrEP in pregnant and post-partum AGYW between the ages of 16 and 24^30^. UNICEF also is conducting a PrEP demonstration project among adolescents in Brazil, South Africa and Thailand to assess facilitators of adherence such as mHealth, peer support, self-improvement models, resilience support, and social media strategies. It is cosponsored by respective ministries of health, increasing the likelihood of generalizability^31^.

## Safety of PrEP

TDF is generally nontoxic, but long-term use is associated with renal, endocrine, and bone toxicities^32^. PrEP trial sub-studies in adolescent and young MSM identified decreased bone mineral density with PrEP use. This was associated with higher parathyroid hormone levels and lower fibroblast growth factor 23 levels, but not with renal abnormalities, suggesting that adolescents may be particularly vulnerable to TDF adverse effects^7,32^. Tenofovir alafenamide (TAF) with FTC (trade name Descovy^®^) may be an alternative to TDF/FTC for PrEP, and phase 3 clinical trials are underway^33^. Looking ahead, an alternative long-acting injectable PrEP containing cabotegravir has been shown to be safe as a long-acting injectable PrEP in phase 2 trials^34^, which could also help to improve adherence.

HIV infection can be acquired with inadequate adherence to PrEP. In these situations the fluctuating serum concentrations of antiretrovirals are not sufficient to prevent acquisition of infection, but they may be present in concentrations that contribute to HIV viral mutations and subsequent antiretroviral resistance. While frequent HIV testing is done to avoid this risk, resistance mutations were seen in 25% of new HIV acquisitions in the treatment group of the Partners PrEP study^9^.

## Adherence

**Figure 1:**
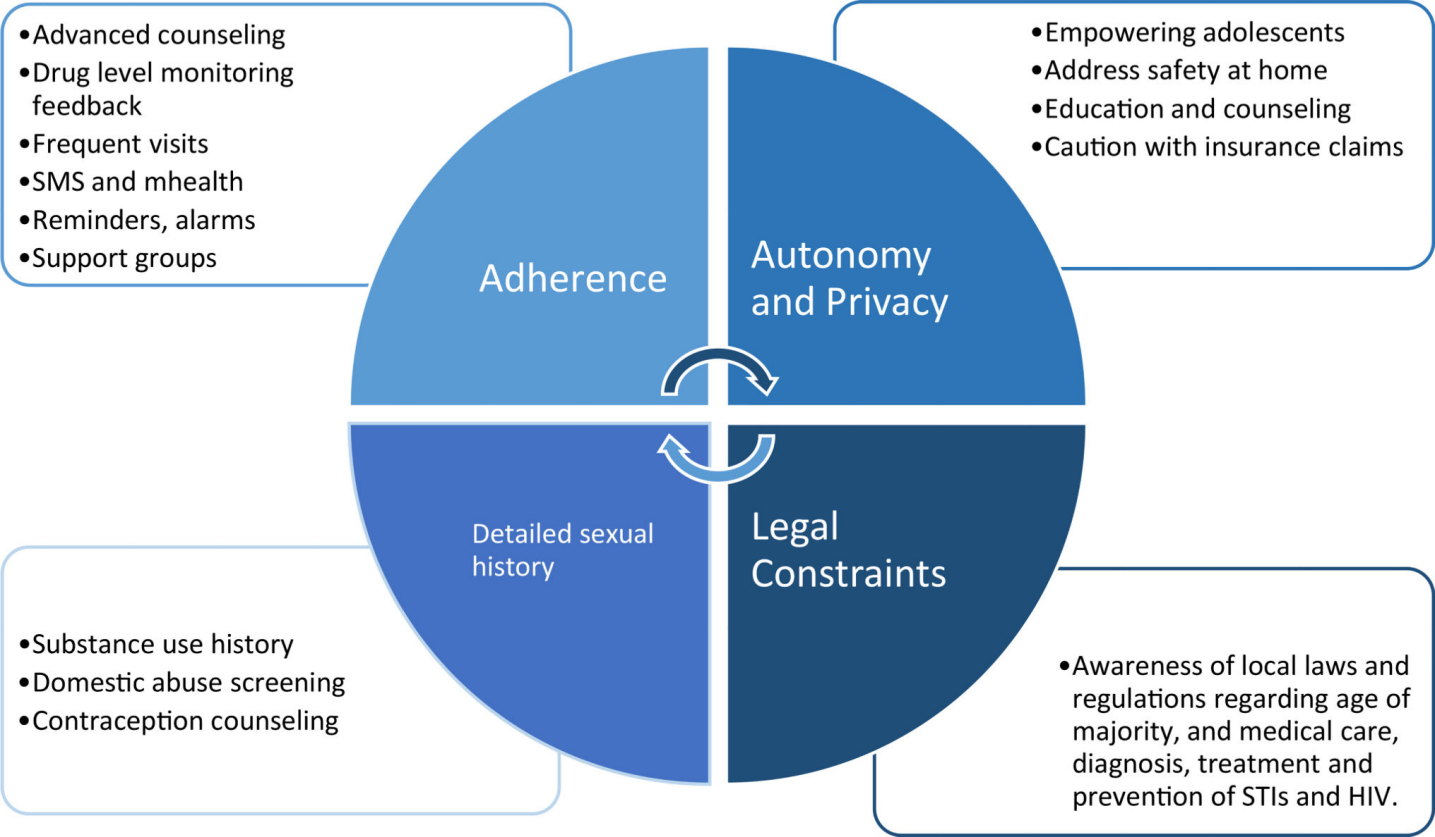
Barriers and facilitators to PrEP for adolescents at high risk for HIV infection.

Adherence is essential to the efficacy of PrEP. Data from ATN-113 suggests that adherence to oral PrEP will also be a major challenge for adolescents, and more frequent clinic visits may be the best first step to improve adherence^7^. Stigma and disapproval from family, partners and peers is a deterrent for many. Forgetfulness, perception of low HIV risk, concerns over side effects, potential toxicities, and cost were all reasons for poor adherence among adults in PrEP clinical trials^35^. Ethnographic factors in each community will present unique adherence barriers, which may be best addressed through peer navigators, support groups, and social networking^36^, though a multi-faceted approach for adolescents is likely necessary (Figure 1).

Specific approaches to supporting PrEP adherence have been evaluated in adults, although adherence facilitators may not be the same for adolescents and further studies are needed to evaluate this. Facilitators to PrEP adherence in adults include reminders, peer support, routines, advanced counseling, drug monitoring feedback and mHealth interventions^37^. Two specific advanced counseling methods, LifeSteps and Next-step, both involve motivational interviewing and problem solving. Through Next-step counseling interviews, used in the iPrEx Open Label Extension^38,39^, the most commonly reported facilitator of PrEP adherence was linking the dose to a daily routine event (86%), and the most common barrier was disruption in routine (37%)^40^. LifeSteps was used in an ancillary study of Partners PrEP and was credited with an 8% increase in adherence^41^. Drug detection feedback for use in adherence counseling has been evaluated qualitatively and was a motivational factor for some^42^. The use of mobile phone applications and text messages has also improved PrEP adherence in adult studies^43^.

## Access to PrEP

While adolescents at high HIV risk generally have positive attitudes towards PrEP^44^, the ability to find PrEP, afford it, and maintain privacy may be challenging. PrEP uptake has been low in the United States, especially in primary care^5,45^. The role of prescribing antiretroviral therapy has historically and primarily resided with the Infectious Disease specialist, and there is reticence among primary care physicians to prescribe PrEP^46^. As of 2015, only one in three primary care physicians had even heard of PrEP^47^. However, providing PrEP does not require formal certification and primary care physicians are in an optimal position to reach the broadest base of high risk patients. A PrEP tool kit targeted at primary care physicians is available through the Sexuality Information and Education Council of the United States (http://www.siecus.org/index.cfm?fuseaction=page.viewPage&pageID=1555). Table 2 offers guidance for initiating PrEP in adolescents.

In the United States, most health insurance companies do cover PrEP. Using parental health insurance may lead to privacy concerns for adolescents seeking PrEP through explanation of benefits letters or other health insurance paperwork. Many other adolescents and youth are uninsured, impeding access to PrEP services^48^. Those in rural areas may find it particularly challenging to access PrEP^49^. An online resource is available to help find PrEP for those without insurance (https://preplocator.org/).

In the United States, the laws pertaining to testing, treatment, and prevention of sexually transmitted infections without parental consent vary by state. Awareness of these laws and regulations are necessary for ethical provision of PrEP, as most minors cannot legally consent to PrEP alone. Emancipated minors, those with children, those in the military, and minors assessed as being “mature” may all be able to consent to PrEP, depending on state law^50^. In South Africa, children ages 12 and older can consent to medical treatment without parental consent if they are able to understand the risks and benefits of the medical treatment^51^. Each country’s regulations regarding privacy, age of consent, payer options and government funding will result in a unique landscape for the provision of PrEP to high risk adolescents.

## Conclusion

The goal of preventing new HIV infections among adolescents is critical. Though there is progress, substantial gaps remain in adolescent HIV prevention research. PrEP is an available and potentially highly effective method of HIV prevention for adolescents at elevated risk, but identifying these adolescents and linking them to PrEP care may be challenging. Additionally, adherence is both essential to PrEP efficacy and particularly difficult for young persons, making strategies to improve adherence an important next step in PrEP public health interventions and research.

## Figures and Tables

**Table 1:** Challenges in PrEP Implementation for High Risk Adolescents Raised by a Youth Panel.

Challenges in PrEP Implementation for High Risk Adolescents
Legal constraints related to age of consent for sexual and reproductive health care
Sexual violence and non-supportive sexual partners
PrEP cost
Discrimination related to sexual orientation, gender, or addiction disorders
Stigma from health service providers, the public, and adolescents’ friends, family and sexual partners
Limited capacity to assess HIV acquisition risk

**Table 2:** Guidance for Initiating PrEP in High Risk Adolescents.

Guidance for Initiating PrEP in High Risk Adolescents	
1.	Anticipatory guidance should be given regarding stigma. Discussions regarding disclosure of PrEP to friends, families and sexual partners are valuable, upon initiation and in follow-up.
2.	Adverse effects of oral TDF-based PrEP must be discussed, including a risk for nephrotoxicity and decreased bone mineral density, both being reversible with discontinuation of PrEP (5).
3.	Side-effects of TDF-FTC must be reviewed, including the “start-up syndrome,” which consists of nausea, mild abdominal pain or headache in the first four weeks of use and only occurs in 10% of people (5).
4.	STI counseling should be given, underscoring the continued risk for other STIs without condom use.
5.	Adolescents should be informed that their risk for new HIV infection is not eliminated, making routine follow up appointments and laboratory testing important.
6.	Patients who are started on PrEP require follow-up every 1–3 months for monitoring for new HIV infection, renal function, pregnancy, and adherence support.
7.	The need for PrEP should be re-evaluated as behaviors and sexual partners change over time.
8.	For adolescents with substance use disorders, counseling on safe injection practices and provision of resources for addiction treatment should be provided.
